# Neurotransmitter and tryptophan metabolite concentration changes in the complete Freund’s adjuvant model of orofacial pain

**DOI:** 10.1186/s10194-020-01105-6

**Published:** 2020-04-21

**Authors:** Edina K. Cseh, Gábor Veres, Tamás Körtési, Helga Polyák, Nikolett Nánási, János Tajti, Árpád Párdutz, Péter Klivényi, László Vécsei, Dénes Zádori

**Affiliations:** 1 Department of Neurology, Interdisciplinary Excellence Center, Faculty of Medicine, Albert Szent-Györgyi Clinical Center, University of Szeged, Semmelweis u. 6, Szeged, H-6725 Hungary; 2 MTA-SZTE Neuroscience Research Group, Szeged, Hungary

**Keywords:** Migraine, CFA model, Orofacial pain, Glutamate, Kynurenic acid

## Abstract

**Background:**

The neurochemical background of the evolution of headache disorders, still remains partially undiscovered. Accordingly, our aim was to further explore the neurochemical profile of Complete Freund’s adjuvant (CFA)-induced orofacial pain, involving finding the shift point regarding small molecule neurotransmitter concentrations changes vs. that of the previously characterized headache-related neuropeptides. The investigated neurotransmitters consisted of glutamate, γ-aminobutyric acid, noradrenalin and serotonin. Furthermore, in light of its influence on glutamatergic neurotransmission, we measured the level of kynurenic acid (KYNA) and its precursors in the kynurenine (KYN) pathway (KP) of tryptophan metabolism.

**Methods:**

The effect of CFA was evaluated in male Sprague Dawley rats. Animals were injected with CFA (1 mg/ml, 50 μl/animal) into the right whisker pad. We applied high-performance liquid chromatography to determine the concentrations of the above-mentioned compounds from the trigeminal nucleus caudalis (TNC) and somatosensory cortex (ssCX) of rats. Furthermore, we measured some of these metabolites from the cerebrospinal fluid and plasma as well. Afterwards, we carried out permutation t-tests as post hoc analysis for pairwise comparison.

**Results:**

Our results demonstrated that 24 h after CFA treatment, the level of glutamate, KYNA and that of its precursor, KYN was still elevated in the TNC, all diminishing by 48 h. In the ssCX, significant concentration increases of KYNA and serotonin were found.

**Conclusion:**

This is the first study assessing neurotransmitter changes in the TNC and ssCX following CFA treatment, confirming the dominant role of glutamate in early pain processing and a compensatory elevation of KYNA with anti-glutamatergic properties. Furthermore, the current findings draw attention to the limited time interval where medications can target the glutamatergic pathways.

## Background

Although the pathomechanism of orofacial pain and headache disorders, is not fully understood [1], the activation and sensitization of the trigeminovascular system (TS) probably takes part in the evolution of symptoms [2–4]. The pathomechanism of these disorders may be further investigated by using animal models with the activation of nociceptive pathways of the TS [1, 3, 5]. The administration of inflammation-inducing substances to the orofacial area can evoke the above-described activation/sensitization of the primary and secondary trigeminal neurons during pain processing [6, 7]. For the induction of this peripheral inflammation, the application of Complete Freund’s adjuvant (CFA) into the whisker pad or the dural parietal surface is a widely used method [6, 8, 9] as it is able to enhance local reaction at the injection site and then to evoke the release of inflammatory cytokines, alongside with hyperalgesia/allodynia on the face via the activation/sensitization of the TS [7]. Regarding the delay of the development of peripheral and central sensitization, indirect data from studies with CFA injection to the paw demonstrated that pain hypersensitivities were observed 24 h after the injection [9–13], whereas data from studies with orofacial CFA model, more precisely from the temporomandibular joint induced inflammation model, suggest that both thermal and mechanical allodynia peak at 24 h as well [14]. The orofacial CFA model has been thoroughly studied regarding gene expression characteristics [6, 15–20]. Recently, in relation to two migraine-related biomarkers, the pituitary adenylate cyclase-activating peptide (PACAP) and calcitonin gene-related peptide (CGRP), their increasing levels were detected starting even 24 h after the administration of CFA in the trigeminal nucleus caudalis (TNC) [9]. However, there are no studies which aimed at the investigation of the small molecule neurotransmitters and neuromodulators and some of their precursors (glutamate (Glu), γ-aminobutyric acid (GABA), setotonin (5-hydroxy-tryptamine; 5-HT), noradrenaline (NA), tryptophan (TRP), kynurenine (KYN), kynurenic acid (KYNA)) in this model with established or presumed role in the development of peripheral and central sensitization during headache. Therefore, there are no data about how the concentration changes of these substances affect the evolution of peripheral and central sensitization. Accordingly, finding the transition point where the dominance of small molecule mediated neurotransmission shifts to that of the PACAP and CGRP mentioned earlier may have significant therapeutic consequences in view of the different targeted approaches.

The primary excitatory neurotransmitter Glu plays an important role in the primary sensory neurotransmission and trigeminal nociception [15, 21, 22]. Accordingly, the alteration of Glu levels in migraine has been widely studied and data consistently show elevated Glu levels in the CSF samples of patients with chronic migraine [23], or migraine with and without aura [24], whereas in plasma samples, the results were not consistent across studies [25–27]. Moreover, similar importance has to be attributed to the changes of the concentration of GABA, the main inhibitory neurotransmitter of the central nervous system (CNS), which is capable of modulating the excitatory pathways [28]. Recently, mainly in light of its influence on glutamatergic neurotransmission, special attention was dedicated to the investigation of the effect of KYNA, a compound of the KYN pathway (KP) of the TRP metabolism [29–34]. KYNA can influence glutamatergic neurotransmission in a complex way [35], i.e., it acts as a competitive antagonist at the N-methyl-D-aspartate (NMDA) receptor [36] and has weak antagonistic effects at the α-amino-3-hydroxy-5-methyl-4-isoxazolepropionic acid (AMPA) and kainate receptors as well [37]. 5-HT, another well-known TRP metabolite, released from serotonergic neurons of the raphe nuclei, exerts modulating effect on TS activation [38–40]. Noradrenaline (NA) may be of interest as well, as noradrenergic neurons project to TNC and may have a role in cluster headache, another primary headache disorder [41, 42].

Based on the observed gradually increasing levels of PACAP and CGRP from 24 h following CFA injection in our previous experiment [9], the aim of the current study was to find the shift point of concentration changes of small molecule neurotransmitters and neuromodulators and the above-mentioned peptides. This may yield substantial information for the selection between different therapeutic paradigms regarding diseases involving the activation of the TS, such as primary headache disorders, including migraine.

## Materials and methods

### Animal experiments and sample collection

**Fig. 1:**
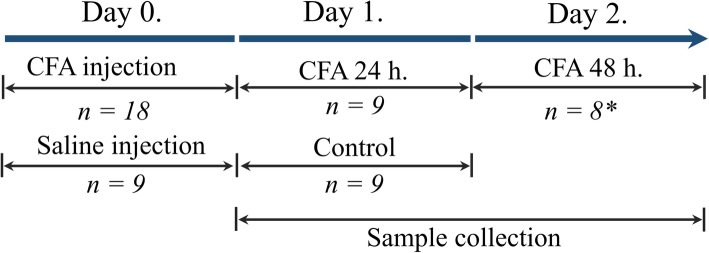
Time-line of the experimental procedure applied in this study. *CFA* Complete Freund’s adjuvant*. n* number of the animals per group. *One animal died in cage after CFA injection

Twenty-seven young adult (10–12 weeks old, 250–300 g) male Sprague-Dawley rats (Charles River Laboratories, Wilmington, MA, USA), were used for the experiments. The animals were bred and maintained under standard laboratory conditions with 12 h–12 h light/dark cycle at 24 ± 1 °C and 50% relative humidity, 3 animals per each home cage in the Laboratory Animal House of the Department of Neurology, University of Szeged. The rats had free access to standard rat chow and water. The experiment was not pre-registered. All experimental procedures performed in this study complied fully with the guidelines of Act 1998/XXVIII of the Hungarian Parliament on Animal Experiments (243/1988) and with the recommendations of the International Association for the Study of Pain and European Communities Council (86/609/ECC). The studies were in harmony with the Ethical Codex of Animal Experiments and were approved by the Ethics Committee of the Faculty of Medicine, University of Szeged, with a permission number of XI./1102/2018. CFA (killed mycobacteria suspended in paraffin oil, 1 mg/ml) was obtained from Sigma-Aldrich (product number: F5881; St. Louis, MO, USA), and 50 μl was administered per animal. We tried to minimalize the use of animals by adopting the key aspects of the 3Rs (Replacement, Reduction and Refinement) [43]. Therefore, the experimental groups were added in a sequential manner, starting from 24 h following CFA administration with 24 h steps till the time point where the proposed alterations diminish. Therefore, no randomization was performed to allocate subjects in the study. By the end of the experiments we had three groups, one control (CO) and two with CFA treatment (Fig. 1). Similar to the previous experiment on PACAP and CGRP in the same model [9], only sham-injected rats processed 24 h following the injection were used as CO, as a pilot study conducted on naïve and sham-injected (processed 24 and 48 h following injection) rats demonstrated that there is no difference in the level of the metabolites of interest, in neither TNC, nor ssCX (*n* = 3 in each group, data not shown). The rats were anesthetized with intraperitoneal 4% chloral hydrate solution mainly based on its safe application (CAS ID: 302–17-0, Sigma-Aldrich, St. Louis, MO, USA; 10 ml/kg body weight dose) in the morning and 50 μl of CFA was injected into the right whisker pad. No other analgesic was applied, otherwise the activation/sensitization phenomena during pain processing, an essential characteristic of the CFA model as well, would have been influenced. Control rats were injected with an equal volume of saline. Cerebrospinal fluid (CSF) was taken from the suboccipital cistern, including the control group (*n* = 9), 24 (*n* = 9) and 48 h (n = 9 initially, finally *n* = 8 as one animal died during the experiment) after injection applying the above-described anesthetic procedure, and following that the animals were perfused transcardially with 200 ml phosphate-buffered saline (PBS). The spinal tap procedures were unsuccessful in 5 occasions and 7 of the CSF samples were excluded from analysis due to contamination with blood. Accordingly, 5–5 samples remained in the CO and CFA 24 h groups, and 4 in the CFA 48 h group for analysis. Therefore, this part of the study focusing at that secondary endpoint was only exploratory due to the low statistical power. Also as a secondary endpoint, blood samples were taken from the left ventricle into ice-cold glass tubes containing disodium ethylenediaminetetraacetate dihydrate (Na_2_EDTA; CAS ID: 194491–31-1 Lach-Ner s.r.o, Neratovice, Czech Republic) and the plasma was separated by centrifugation (1170 g for 10 min at 4 °C). Following decapitation two different brain structures, the TNC and the somatosensory cortex (ssCX) were dissected for the assessment of the targeted primary endpoints. In each case both right and left sided samples were separately removed on ice and stored at − 80 °C until further use.

### Instruments and chromatographic conditions

Validated high performance liquid chromatography (HPLC) measurements were performed by an Agilent 1100 HPLC system (Santa Clara, CA, USA), coupled with UV detector (UVD), fluorescence detector (FLD) and electrochemical detector (ECD). The chromatographic separations were carried out with validated methods comprehensively described elsewhere [44–46]. Prior to all measurements, during the tissue weighting or plasma/CSF precipitation process, all samples were relabeled, and a blind study was conducted, i.e., the experimenter who did the HPLC measurements was not aware of which samples were part of CO or 24 h groups. Moreover, Eppendorf tubes were randomly assigned for measurements and when the 48 h group was measured, the same systematic randomization was applied. The purity of all standards and solutions were analytical grade or HPLC grade and they were acquired from Sigma-Aldrich, St. Louis, MO, USA, except the fluorescent internal standard used for the TRP method which was synthesized at the Department of Pharmaceutical Chemistry, University of Szeged, as detailed elsewhere [44]. Briefly, the brain regions were homogenized in 0.5 M perchloric acid (PCA), at 1:5 w/v containing internal standards (ISs, 3-nitro-L-tyrosine and 4-hydroxyquinazoline-2-carboxylic acid, the latter custom-made material will be shared upon reasonable request) applied in the measurement of TRP metabolites [44] utilizing both UVD and FLD. After centrifugation the supernatant was collected and first used for the TRP metabolite measurement. The remaining supernatant was aliquoted in two further parts and were kept at − 80 °C until further analyses. 150 μl from it was applied for the determination of NA concentration by ECD [45], with addition of 10 μl solution of the corresponding IS, 2,3-dihydroxybenzoic acid. For the measurement of Glu and GABA, another 100 μl was diluted to 1:100 v/v with distilled water and 100 μl of this dilution was derivatized with 100 μl solution containing o-phthaldialdehyde and 3-mercaptopropionic acid in borate buffer and further diluted with 50 μl distilled water containing the corresponding IS, homoserine, used for this method applying FLD [46].

For the measurement of the TRP metabolites from the CSF, the method described before [44] was applied, with a slight modification. Briefly, during sample preparation, we used a dilution of 5:6 v/v, with the final concentration of PCA at 0.5 M, with the above described ISs, but only 35 μL of the sample was injected. Furthermore, a linearity study was conducted for rat CSF samples to determine limit of detection (LOD) and limit of quantitation (LOQ) values, because the cited article contains data only for human CSF. Accordingly, the LOD and LOQ values for rat CSF were 31.1 and 102 nM for TRP, 107 and 702 nM for KYN and 1.04 and 3.45 nM for KYNA, respectively, whereas 5-HT was undetectable in each case. Regarding Glu and GABA, the initial amount of mobile phase A applied for the brain samples was 95%, but for CSF samples it was changed to 93%, as coelution was observed under the initial circumstances. The ratios applied for the CSF sample preparation (1:1:0.5 = sample: derivatization solution: IS) remained the same, similar to brain supernatants [46]. Due to low sample amount we omitted the determination of NA levels from CSF.

With regard to plasma samples we measured the levels of TRP metabolites as described in [44]. Glu, GABA and NA concentrations from plasma samples were not assessed because we were only interested in their role as a neurotransmitter.

As for the plasma samples, the LOD and LOQ values were 0.102 μM and 0.308 μM for TRP, 0.027 and 0.083 μM for KYN and 1.23 and 3.72 nM for KYNA, respectively. In each case, the 5-HT levels from plasma samples were undetectable.

### Statistical analyses

All statistical calculations were performed with the use of the freely available R software 3.5.3 (R Development Core Team). The distribution of our data population was not determined as the applied statistical tests do not need assumptions regarding the distribution of underlying data. Accordingly, first we performed the Levene test to assess the homogeneity of variances. As the variances were equal, we performed a general independence test for two sets of variables measured on arbitrary scales, where the reference distribution was approximative based on the Monte-Carlo method. Afterwards, we carried out permutation t-tests as post hoc analysis for pairwise comparison. Permutations were applied via the Monte-Carlo method (10,000 random permutations) and Type I errors from multiple comparisons were controlled with false discovery rate. No test for outliers was conducted. With the key aspects of 3Rs in mind [43] we tried to keep the sample size as low as we can based on experiences from previous experiments ([47]: 8 and 12/group; [48]: 6/group; [49]: 6/group; [50]: 6/group; [51]: 8/group; [52]: 6 and 7/group; [53]: 6/group). For every statistically significant result, we calculated the corresponding effect size (Cohen’s d in this case) and based on its value, we decided whether the increase of sample size is necessary or not. The manuscript contains the final effect sizes.

## Results

### Concentration levels of the assessed compounds in the TNC and ssCX

First of all, both contralateral and ipsilateral CNS regions were measured separately, but we did not find significant differences in concentrations of any of the metabolites between the two sides, so the coherent data were pooled for further analysis. Therefore, the concentration values presented in Table 1 demonstrate the mean values of the two analyzed sides of each CNS regions.

**Table 1:** Concentration levels of the measured metabolites in the analyzed brain regions

	Control group (*n =* 9)	CFA 24 h (*n* = 9)	CFA 48 h (*n* = 8^†^)
Trigeminal nucleus caudalis (TNC)			
Glu (μg/g ww)	684 (644–746)	772^*,#^ (742–859)	731 (687–745)
GABA (μg/g ww)	167 (154–187)	180 (174–235)	167 (164–171)
TRP (nmol/g ww)	20.3 (19.2–22.4)	20.3 (18.2–24.5)	19.4 (17.7–20.8)
KYN (nmol/g ww)	0.656 (0.428–0.671)	0.876^*,#^ (0.830–1.13)	0.532 (0.480–0.597)
KYNA (pmol/g ww)	22.8 (21.2–24.2)	52.6^**,#^ (34.6–72.3)	25.8 (21.9–28.8)
5-HT (pmol/g ww)	2991 (2917–3333)	2841 (2629–3425)	3315 (3088–3438)
NA (μg/g ww)	0.328 (0.320–0.343)	0.352 (0.328–0.388)	0.348 (0.324–0.366)
Somatosensory cortex (ssCX)			
Glu (μg/g ww)	1178 (1082–1290)	1269 (1206–1397)	1152 (1052–1287)
GABA (μg/g ww)	215 (207–218)	230 (217–251)	199 (178–211)
TRP (nmol/g ww)	20.6 (17.8–23.5)	22.6 (21.5–23.7)	21.6 (20.9–22.7)
KYN (nmol/g ww)	0.824 (0.743–0.970)	0.974 (0.714–1.15)	0.616 (0.552–0.663)
KYNA (pmol/g ww)	16.2 (9.70–18.8)	27.3^*,#^ (17.3–39.3)	9.73 (7.01–12.8)
5-HT (pmol/g ww)	2547 (1665–2677)	2271^#^ (2166–2527)	2885 (2653–3172)
NA (μg/g ww)	0.840 (0.192–0.853)	0.754 (0.142–0.934)	0.886 (0.556–0.974)

Results are shown as median (1st-3rd quartile). ^†^One animal died in cage after CFA injection. * *p* < 0.05 vs. CO, ** *p* < 0.01 vs. CO, # *p* < 0.05 vs. 48 h, *5-HT* serotonin, *CFA* Complete Freund’s adjuvant*, GABA* gamma-aminobutyric acid, *Glu* glutamate, *KYN* kynurenine, *KYNA* kynurenic acid, *n* number of the animals per group, *NA* noradrenaline, *TRP* tryptophan, *ww* wet weight

**Fig. 2:**
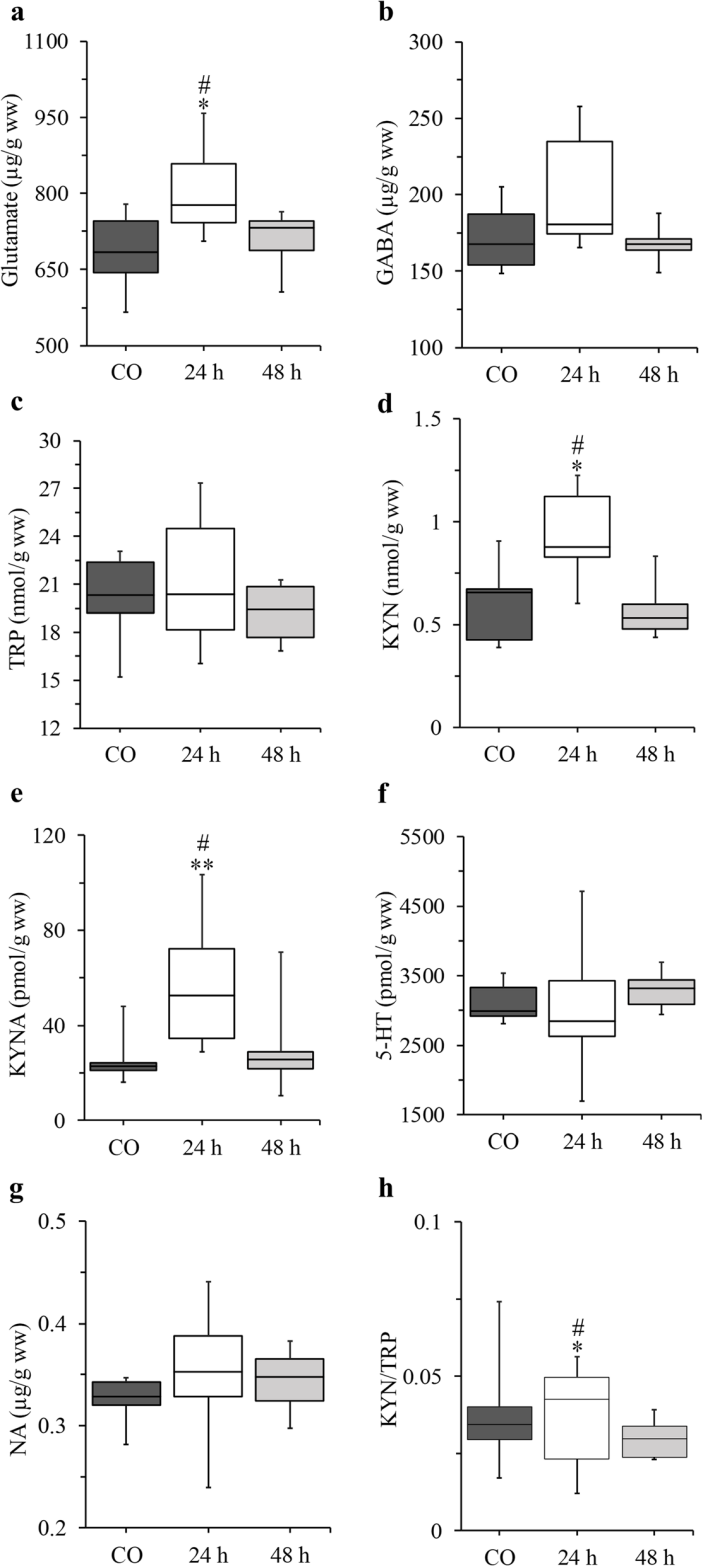
Concentration changes in glutamate (**a**), γ-aminobutyric acid (**b**), tryptophan (**c**), kynurenine (**d**), kynurenic acid (**e**), serotonin (**f**), noradrenaline (**g**) and changes in kynurenine/tryptophan ratio (**h**) in the TNC. * *p* < 0.05 vs. CO, ** *p* < 0.01 vs. CO, # *p* < 0.05 vs. 48 h. *n* = 9 in the control and 24 h groups and *n* = 8 in the 48 h group. The boxplots are displayed as the intervals between the 1st and 3rd quartiles presenting the median values as well. *24 and 48 h* CFA treated groups*, 5-HT* serotonin, *CO* control*, GABA* γ-aminobutyric acid, *KYN* kynurenine, *KYNA* kynurenic acid, *n* number of the animals per group, *NA* noradrenaline, *TRP* tryptophan, *TNC* trigeminal nucleus caudalis, *ww* wet weight

Regarding TNC, pairwise permutation t-tests following the independence tests revealed a significant elevation in the concentration of Glu (*p* = 0.0319, Cohen’s d = 1.49), KYN (*p* = 0.0123, Cohen’s d = 1.58) and KYNA (*p* = 0.0098, Cohen’s d = 1.92) 24 h following CFA injection compared to the controls and a significant decrease could be observed in Glu (*p* = 0.0357, Cohen’s d = 1.29), KYN (*p* = 0.0123, Cohen’s d = 1.85) and KYNA (*p* = 0.0263, Cohen’s d = 1.39) levels by 48 h compared to the 24 h group, whereas there was no difference between the control and 48 h groups (Table 1, Fig. 2).

**Fig. 3:**
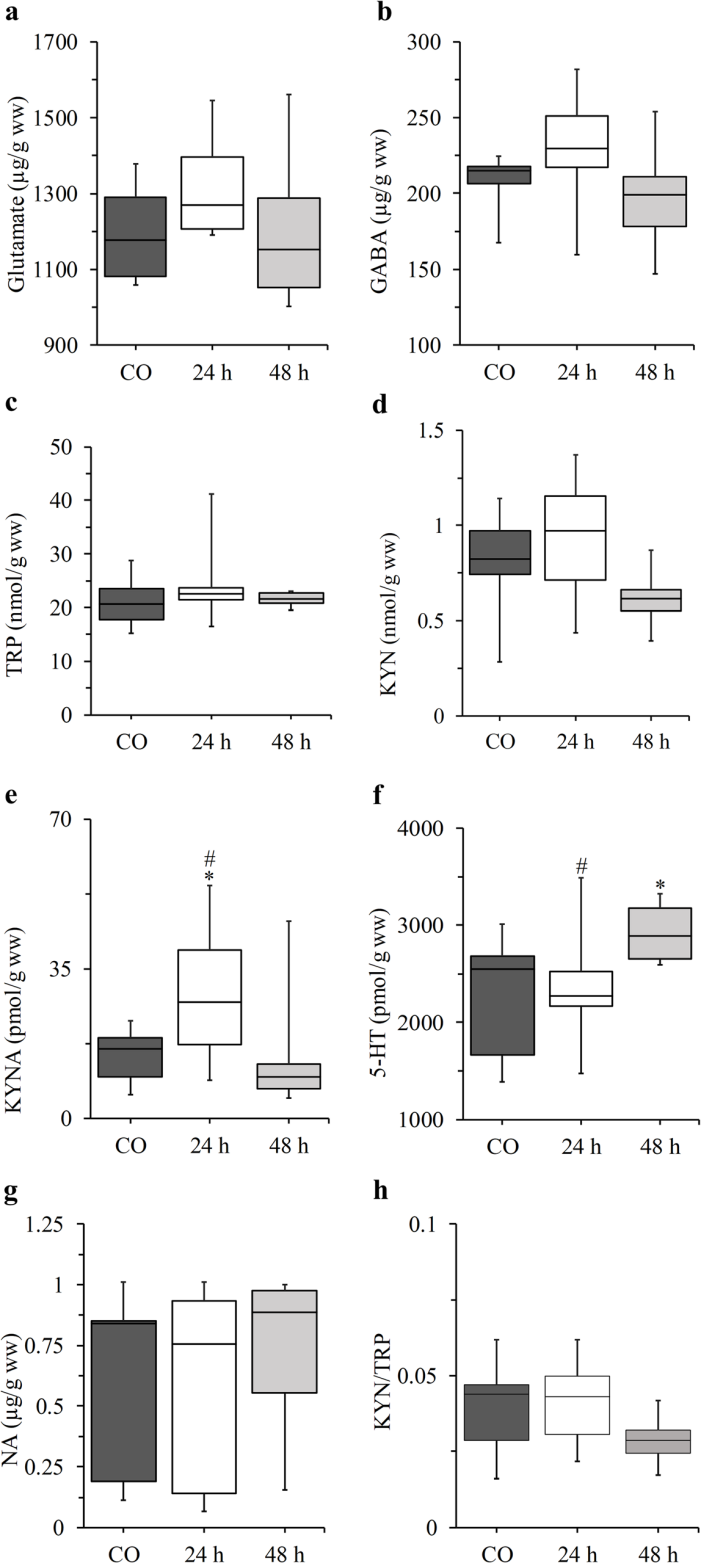
Concentration changes in glutamate (**a**), γ-aminobutyric acid (**b**), tryptophan (**c**), kynurenine (**d**), kynurenic acid (**e**), serotonin (**f**), noradrenaline (**g**) and changes in kynurenine/tryptophan ratio (**h**) in the somatosensory cortex. * *p* < 0.05 vs. CO, # *p* < 0.05 vs. 48 h. n = 9 in the control and 24 h groups and *n* = 8 in the 48 h group. The boxplots are displayed as the intervals between the 1st and 3rd quartiles presenting the median values as well. *24 and 48 h* CFA treated groups*, 5-HT* serotonin, *CO* control*, GABA* γ-aminobutyric acid, *KYN* kynurenine, *KYNA* kynurenic acid, *n* number of the animals per group, *NA* noradrenaline, *TRP* tryptophan, *TNC* trigeminal nucleus caudalis, *ww* wet weight

Regarding ssCX samples, an elevation in KYNA concentration (*p* = 0.0237, Cohen’s d = 1.36) could be observed 24 h following CFA administration, followed by a significant decrease by 48 h (*p* = 0.0173, Cohen’s d = 1.80) and there was no difference between control and 48 h groups. Furthermore, in the ssCX, there was a significant increase in 5-HT levels in the 48 h group compared to the controls (*p* = 0.0479, Cohen’s d = 1.21) and to the 24 h group (*p* = 0.0479, Cohen’s d = 1.20; Table 1, Fig. 3).

We calculated the KYN/TRP and KYNA/KYN ratios as well. The KYN/TRP ratio was significantly elevated in the 24 h group compared to the controls (*p* = 0.0419, Cohen’s d = 1.19) or to the 48 h group (*p* = 0.0419, Cohen’s d = 1.35; Table 1, Fig. 2). With regard to the KYNA/KYN ratio, there was no difference in any of the investigated biological matrices (data no shown).

### CSF and plasma samples

Regarding CSF samples, TRP metabolites, Glu and GABA were measured. We found no significant alterations in the CSF, however, the power of the statistical tests in this case is low due to low case number (*n* = 5, 5, 4 for control, 24 h and 48 h groups, respectively) and the concentration values of KYN in the control and CFA treated 48 h groups were below LOD (0.107 μM), except one case from each group (for more details, see Additional file 1, Table S1; due to the low amount of 5-HT in the CSF samples, we could not quantify it, as the values were below LOD, LOD = 0.0274 μM). In case of plasma samples, only the TRP metabolites were measured, and no significant differences were observed (for more details, see Additional file 2, Table S2).

## Discussion

Headache is one of the most common neurological disorders and it is one of the leading causes of health-related problems worldwide. In 2010, tension type headache and migraine were the second and third most prevalent conditions in the world, respectively, according to the Global Burden of Disease (GBD) study [54, 55]. Furthermore, the GBD study in 2015 established that headache is responsible [56] for more disability adjusted life years than all other neurological disorders in combination.

The treatment of primary headache disorders is challenging, requiring both acute and preventive therapeutic measures [57, 58]. The preventive treatment aims to reduce the frequency, severity and duration of headaches, and to avoid medication-overuse headache. The efficacy of the currently applied drugs is not always satisfactory and the contraindications and side-effects often limit the options of the physician [59, 60]. Therefore, there is a constant need to study and develop new molecules.

### Glutamate and pain

Peripheral and central sensitization manifest mainly in forms of hyperalgesia and allodynia. The activation of the peripheral terminals of the nociceptors is responsible for Glu release at central sites with the activation of ionotropic and metabotropic Glu receptors [61]. This process was demonstrated not only in preclinical studies [62–64], but in patients with headache as well [23, 24]. Accordingly, the role of glutamatergic pathways in association with different types of pain is well established [65] and several antagonists of ionotropic glutamate receptors were investigated and found to be effective to decrease nociceptive transmission [66]. However, they had severe side effects, and therefore, the interest in this direction of research diminished [67, 68]. Nevertheless, ketamine, an NMDA receptor antagonist, is so far the only promising option in the treatment of severe or long-lasting migraine aura [69], and tezampanel, which acts on the AMPA and kainate subtypes of ionotropic Glu receptors [70], has also shown promising results in acute migraine therapy [71].

### Tryptophan metabolism and pain

It has been already demonstrated that the level of KYNA and some other KP metabolites are altered in migraine and cluster headache patients as well: there are significant reductions in the serum levels of KYN, KYNA, 3-hydroxy-kynurenine, 3-hydroxy-anthranilic acid and quinolinic acid, whereas concentrations of TRP and anthranilic acid were significantly increased [72, 73]. KYNA as an endogenous NMDA receptor antagonist, is a molecule of interest for CNS drug development in case of several neurological conditions [74], but due to its poor ability to cross the blood-brain barrier (BBB) and its rapid clearance from the body [75], its application for most CNS-related alterations is limited, and therefore several KYNA analogs were synthetized [76–79]. However, the first order neuron of pain processing is located outside the BBB [80], so KYNA itself may have therapeutic potential as well. Accordingly, the antinociceptive properties of KYNA were proved in animal models of pain [29, 81]. Furthermore, some of the developed analogs also displayed promising results in different animal models of headache [31, 82–85]. In an earlier study we investigated two KYNA analogs where both of them proved to be effective in the formalin model of trigeminal pain [84]. However, one of them was more effective than the other and according to our analyses the better performing compound caused a more pronounced elevation of KYNA concentration on the periphery, whereas in the CNS the concentrations of KYNA were similar. Based on these results we hypothesized that the peripheral elevation of KYNA may be enough to exert beneficial effects on pain processing and targeting this component could provide an option to pharmaceutical drug design without the obligation of good penetration through the BBB.

Elevated Glu concentration in the TNC of CFA-treated rats, demonstrated by the current study, is accompanied by increased KYN and KYNA levels, which may serve as a feedback mechanism to the sensitization process caused by Glu. This hypothesis is supported by the above-mentioned findings [72, 73] that decreased KP metabolite levels are associated with those headache disorders, where increased NMDA receptor activation may play a crucial role. These results may have a great importance especially in light of the finding that the slightly, but not significantly elevated GABA level may not be enough to counterbalance the effects of increased Glu levels. With regard to 5-HT, its cortical elevation by 48 h may serve as a feedback inhibitory response as well to ameliorate the activation of the trigeminovascular pathway [86].

The current study draws attention to the limited time interval for therapies targeting glutamatergic pathways as well, as based on our previous experiments, a clear shift to dominantly peptide-mediated pain processing can be seen even from 24 h after CFA application [9]. This time point corresponds to the onset of peripheral and central sensitization of the TS as well in this model [10, 11, 14]. At this stage, mainly novel antibody-based therapies may come into account [87–90]. With regard to these novel therapies, the focus of attention is on monoclonal antibodies targeting the CGRP pathway for the prophylactic treatment of migraine. Currently, four of these antibodies are in clinical trials (eptinezumab, galcanezumab, fremanezumab, erenumab) with promising results. However, the cost of these therapies is considerably higher than that of acute phase treatments.

## Conclusion

This is the first study assessing small molecule neurotransmitter changes in the TNC and ssCX following CFA treatment, confirming a dominant role of glutamate in early pain processing and a compensatory elevation of KYNA with anti-glutamatergic properties. The time interval for the intervention targeting the glutamatergic system is presumed to be limited to the first 24 h. The results of our previous therapeutic studies with KYNA or with its analogs strongly support this theory.

## Supplementary information

**Additional file 1: Table S1.** Concentration levels of the measured metabolites in the cerebrospinal fluid.

**Additional file 2: Table S2.** Concentration levels of the measured metabolites in the plasma samples.

## Data Availability

The authors made available all of their data and materials on request.

## Abbreviations

5-HT: 5-hydroxy tryptamine (serotonin)
AMPA: α-amino-3-hydroxy-5-methyl-4-isoxazolepropionic acid
CFA: Complete Freund’s adjuvant
CNS: Central nervous system
CGRP: Calcitonin gene-related peptide
CO: Control group
CSF: Cerebrospinal fluid
ECD: Electrochemical detector
FLD: Fluorescence detector
ssCX: Somatosensory cortex
GABA: γ-aminobutyric acid
GBD: Global Burden of Disease
Glu: Glutamate
HPLC: High performance liquid chromatography
IS: Internal standard
KYN: Kynurenine
KYNA: Kynurenic acid
LOD: Limit of detection
LOQ: Limit of quantitation
NA: Noradrenaline
Na_2_EDTA: Disodium ethylenediaminetetraacetic acid
NMDA: N-methyl-D-aspartate
PACAP: Pituitary adenylate cyclase-activating peptide
PCA: Perchloric acid
TNC: Trigeminal nucleus caudalis
TRP: Tryptophan
TS: Trigeminovascular system
TTH: Tension-type headache
UVD: Ultraviolet detector

## References

[1] Brennan KC, Pietrobon D (2018). A systems neuroscience approach to migraine. Neuron.

[2] Noseda R, Burstein R (2013). Migraine pathophysiology: anatomy of the trigeminovascular pathway and associated neurological symptoms, CSD, sensitization and modulation of pain. Pain.

[3] Harriott AM, Strother LC, Vila-Pueyo M, Holland PR (2019). Animal models of migraine and experimental techniques used to examine trigeminal sensory processing. J Headache Pain.

[4] Romero-Reyes M, Uyanik JM (2014). Orofacial pain management: current perspectives. J Pain Res.

[5] Tajti J, Párdutz A, Vámos E, Tuka B, Kuris A, Bohár Z (2011). Migraine is a neuronal disease. J Neural Transm (Vienna).

[6] Aczél T, Kun J, Szőke É, Rauch T, Junttila S, Gyenesei A (2018). Transcriptional alterations in the trigeminal ganglia, nucleus and peripheral blood mononuclear cells in a rat orofacial pain model. Front Mol Neurosci.

[7] Iwata K, Takeda M, Oh SB, Shinoda M. Neurophysiology of orofacial pain. In: Farah CS, Balasubramaniam R, McCullough MJ, editors. Contemporary Oral Medicine, Springer International Publishing; 2017. p. 1–23. doi: 10.1007/978-3-319-28100-1_8-1.

[8] Lukács M, Haanes KA, Majláth Z, Tajti J, Vécsei L, Warfvinge K (2015). Dural administration of inflammatory soup or complete Freund’s adjuvant induces activation and inflammatory response in the rat trigeminal ganglion. J Headache Pain.

[9] Körtési T, Tuka B, Nyári A, Vécsei L, Tajti J (2019). The effect of orofacial complete Freund’s adjuvant treatment on the expression of migraine-related molecules. J Headache Pain.

[10] Kopach O, Viatchenko-Karpinski V, Belan P, Voitenko N (2012). Development of inflammation-induced hyperalgesia and allodynia is associated with the upregulation of extrasynaptic AMPA receptors in tonically firing lamina II dorsal horn neurons. Front Physiol.

[11] Park JS, Yaster M, Guan X, Xu JT, Shih MH, Guan Y (2008). Role of spinal cord alpha-amino-3-hydroxy-5-methyl-4-isoxazolepropionic acid receptors in complete Freund’s adjuvant-induced inflammatory pain. Mol Pain.

[12] Park JS, Voitenko N, Petralia RS, Guan X, Xu JT, Steinberg JP (2009). Persistent inflammation induces GluR2 internalization via NMDA receptor-triggered PKC activation in dorsal horn neurons. J Neurosci.

[13] Zhang B, Tao F, Liaw WJ, Bredt DS, Johns RA, Tao YX (2003). Effect of knock down of spinal cord PSD-93/chapsin-110 on persistent pain induced by complete Freund’s adjuvant and peripheral nerve injury. Pain.

[14] Imbe H, Iwata K, Zhou QQ, Zou S, Dubner R, Ren K (2001). Orofacial deep and cutaneous tissue inflammation and trigeminal neuronal activation. Implications for persistent temporomandibular pain. Cells Tissues Organs.

[15] Okumura M, Iwata K, Yasuda K, Inoue K, Shinoda M, Honda K (2010). Alternation of gene expression in trigeminal ganglion neurons following complete Freund’s adjuvant or capsaicin injection into the rat face. J Mol Neurosci.

[16] Chung MK, Park J, Asgar J, Ro JY (2016). Transcriptome analysis of trigeminal ganglia following masseter muscle inflammation in rats. Mol Pain.

[17] Puehler W, Rittner HL, Mousa SA, Brack A, Krause H, Stein C (2006). Interleukin-1 beta contributes to the upregulation of kappa opioid receptor mrna in dorsal root ganglia in response to peripheral inflammation. Neuroscience.

[18] Wu SX, Zhu M, Wang W, Wang YY, Li YQ, Yew DT (2001). Changes of the expression of 5-HT receptor subtype mRNAs in rat dorsal root ganglion by complete Freund’s adjuvant-induced inflammation. Neurosci Lett.

[19] Luo H, Cheng J, Han JS, Wan Y (2004). Change of vanilloid receptor 1 expression in dorsal root ganglion and spinal dorsal horn during inflammatory nociception induced by complete Freund’s adjuvant in rats. Neuroreport.

[20] Demartini C, Tassorelli C, Zanaboni AM, Tonsi G, Francesconi O, Nativi C (2017). The role of the transient receptor potential ankyrin type-1 (TRPA1) channel in migraine pain: evaluation in an animal model. J Headache Pain.

[21] Takeda M, Tanimoto T, Kadoi J, Nasu M, Takahashi M, Kitagawa J (2007). Enhanced excitability of nociceptive trigeminal ganglion neurons by satellite glial cytokine following peripheral inflammation. Pain.

[22] Krzyzanowska A, Avendaño C (2012). Behavioral testing in rodent models of orofacial neuropathic and inflammatory pain. Brain Behav.

[23] Peres MFP, Zukerman E, Senne Soares CA, Alonso EO, Santos BFC, Faulhaber MHW (2004). Cerebrospinal fluid glutamate levels in chronic migraine. Cephalalgia.

[24] Martínez F, Castillo J, Rodríguez JR, Leira R, Noya M (1993). Neuroexcitatory amino acid levels in plasma and cerebrospinal fluid during migraine attacks. Cephalalgia.

[25] Ferrari MD, Odink J, Bos KD, Malessy MJ, Bruyn GW (1990). Neuroexcitatory plasma amino acids are elevated in migraine. Neurology.

[26] Cananzi AR, D’Andrea G, Perini F, Zamberlan F, Welch KM (1995). Platelet and plasma levels of glutamate and glutamine in migraine with and without aura. Cephalalgia.

[27] Campos F, Sobrino T, Pérez-Mato M, Rodríguez-Osorio X, Leira R, Blanco M (2013). Glutamate oxaloacetate transaminase: a new key in the dysregulation of glutamate in migraine patients. Cephalalgia.

[28] Guerriero RM, Giza CC, Rotenberg A (2015). Glutamate and GABA imbalance following traumatic brain injury. Curr Neurol Neurosci Rep.

[29] Knyihár-Csillik E, Chadaide Z, Okuno E, Krisztin-Péva B, Toldi J, Varga C (2004). Kynurenine aminotransferase in the supratentorial dura mater of the rat: effect of stimulation of the trigeminal ganglion. Exp Neurol.

[30] Vámos E, Párdutz Á, Varga H, Bohár Z, Tajti J, Fülöp F (2009). L-kynurenine combined with probenecid and the novel synthetic kynurenic acid derivative attenuate nitroglycerin-induced nNOS in the rat caudal trigeminal nucleus. Neuropharmacology.

[31] Vámos E, Fejes A, Koch J, Tajti J, Fülöp F, Toldi J (2010). Kynurenate derivative attenuates the nitroglycerin-induced CamKIIα and CGRP expression changes. Headache.

[32] Chauvel V, Vamos E, Pardutz A, Vecsei L, Schoenen J, Multon S (2012). Effect of systemic kynurenine on cortical spreading depression and its modulation by sex hormones in rat. Exp Neurol.

[33] Párdutz Á, Fejes A, Bohár Z, Tar L, Toldi J, Vécsei L (2012). Kynurenines and headache. J Neural Transm.

[34] Körtési T, Tuka B, Tajti J, Bagoly T, Fülöp F, Helyes Z (2017). Kynurenic acid inhibits the electrical stimulation induced elevated pituitary Adenylate Cyclase-activating polypeptide expression in the TNC. Front Neurol.

[35] Zádori D, Klivényi P, Plangár I, Toldi J, Vécsei L (2011). Endogenous neuroprotection in chronic neurodegenerative disorders: with particular regard to the kynurenines. J Cell Mol Med.

[36] Kessler M, Terramani T, Lynch G, Baudry M (1989). A glycine site associated with N-methyl-D-aspartic acid receptors: characterization and identification of a new class of antagonists. J Neurochem.

[37] Birch PJ, Grossman CJ, Hayes AG (1988). Kynurenate and FG9041 have both competitive and non-competitive antagonist actions at excitatory amino acid receptors. Eur J Pharmacol.

[38] Deen M, Hansen HD, Hougaard A, Nørgaard M, Eiberg H, Lehel S (2018). High brain serotonin levels in migraine between attacks: a 5-HT4 receptor binding PET study. Neuroimage Clin.

[39] Aggarwal M, Puri V, Puri S (2012). Serotonin and CGRP in migraine. Ann Neurosci.

[40] Varga H, Párdutz A, Tajti J, Vécsei L, Schoenen J (2006). The modulatory effect of estrogen on the caudal trigeminal nucleus of the rat in an animal model of migraine. Ideggyogy Sz.

[41] Bussone G (2008). Cluster headache: from treatment to pathophysiology. Neurol Sci.

[42] Benarroch EE (2018). Locus coeruleus. Cell Tissue Res.

[43] Prescott MJ, Lidster K (2017). Improving quality of science through better animal welfare: the NC3Rs strategy. Lab Anim (NY).

[44] Cseh EK, Veres G, Szentirmai M, Nánási N, Szatmári I, Fülöp F (2019). HPLC method for the assessment of tryptophan metabolism utilizing separate internal standard for each detector. Anal Biochem.

[45] Nánási N, Hadady L, Cseh E, Veres G, Klivényi P, Vécsei L, Tünde A, István I (2018). Development and validation of high performance liquid chromatography method for the measurements of biogenic amines. Proceedings of the 24th International Symposium on Analytical and Environmental Problems.

[46] Veres G, Tellér A, Martos D, Szatmari I, Kiss L, Vécsei L, Tünde A, István I (2019). Determination of glutamate and GABA from rat central nervous system samples with HPLC utilizing fluorescent detection. Proceedings of the 25th International Symposium on Analytical and Environmental Problems.

[47] Pawlak D, Tankiewicz A, Buczko W (2001). Kynurenine and its metabolites in the rat with experimental renal insufficiency. J Physiol Pharmacol.

[48] Ceresoli-Borroni G, Rassoulpour A, Wu HQ, Guidetti P, Schwarcz R (2006). Chronic neuroleptic treatment reduces endogenous kynurenic acid levels in rat brain. J Neural Transm (Vienna).

[49] Kucharewicz I, Kasacka I, Pawlak D, Tankiewicz-Kwedlo A, Mroczko B, Buczko W (2008). The concentration of kynurenine in rat model of asthma. Folia Histochem Cytobiol.

[50] Sultana N, Arayne MS, Khan MM, Saleem DM, Mirza AZ (2012). Determination of tryptophan in raw materials, rat brain and human plasma by RP-HPLC technique. J Chromatogr Sci.

[51] Zagajewski J, Drozdowicz D, Brzozowska I, Hubalewska-Mazgaj M, Stelmaszynska T, Laidler PM (2012). Conversion L-tryptophan to melatonin in the gastrointestinal tract: the new high performance liquid chromatography method enabling simultaneous determination of six metabolites of L-tryptophan by native fluorescence and UV-VIS detection. J Physiol Pharmacol.

[52] Samavati R, Zádor F, Szűcs E, Tuka B, Martos D, Veres G (2017). Kynurenic acid and its analogue can alter the opioid receptor G-protein signaling after acute treatment via NMDA receptor in rat cortex and striatum. J Neurol Sci.

[53] Wu H-Q, Guidetti P, Goodman JH, Varasi M, Ceresoli-Borroni G, Speciale C (2000). Kynurenergic manipulations influence excitatory synaptic function and excitotoxic vulnerability in the rat hippocampus in vivo. Neuroscience.

[54] Saylor D, Steiner TJ (2018). The global burden of headache. Semin Neurol.

[55] Abraham J, Ackerman I, Aggarwal R, Ahn SY, Ali MK, Alvarado M (2012). Years lived with disability (YLDs) for 1160 sequelae of 289 diseases and injuries 1990-2010: a systematic analysis for the global burden of disease study 2010. Lancet.

[56] GBD 2015 DALYs and HALE Collaborators (2016). Global, regional, and national disability-adjusted life-years (DALYs) for 315 diseases and injuries and healthy life expectancy (HALE), 1990–2015: a systematic analysis for the Global Burden of Disease Study 2015. Lancet.

[57] Schuster NM, Rapoport AM (2016). New strategies for the treatment and prevention of primary headache disorders. Nat Rev Neurol.

[58] American Headache Society (2019). The American headache society position statement on integrating new migraine treatments into clinical practice. Headache.

[59] Diener HC, Charles A, Goadsby PJ, Holle D (2015). New therapeutic approaches for the prevention and treatment of migraine. Lancet Neurol.

[60] Obermann M, Holle D, Naegel S, Burmeister J, Diener HC (2015). Pharmacotherapy options for cluster headache. Expert Opin Pharmacother.

[61] Sarchielli P, Di Filippo M, Nardi K, Calabresi P (2007). Sensitization, glutamate, and the link between migraine and fibromyalgia. Curr Pain Headache Rep.

[62] Bereiter DA, Benetti AP (1996). Excitatory amino release within spinal trigeminal nucleus after mustard oil injection into the temporomandibular joint region of the rat. Pain.

[63] Lukács M, Warfvinge K, Tajti J, Fülöp F, Toldi J, Vécsei L (2017). Topical dura mater application of CFA induces enhanced expression of c-fos and glutamate in rat trigeminal nucleus caudalis: attenuated by KYNA derivate (SZR72). J Headache Pain.

[64] Oshinsky ML, Luo J (2006). Neurochemistry of trigeminal activation in an animal model of migraine. Headache.

[65] Osikowicz M, Mika J, Przewlocka B (2013). The glutamatergic system as a target for neuropathic pain relief. Exp Physiol.

[66] Bleakman D, Alt A, Nisenbaum ES (2006). Glutamate receptors and pain. Semin Cell Dev Biol.

[67] Eide K, Stubhaug A, Oye I, Breivik H (1995). Continuous subcutaneous administration of the N-methyl-D-aspartic acid (NMDA) receptor antagonist ketamine in the treatment of post-herpetic neuralgia. Pain.

[68] Jevtovic-Todorovic V, Wozniak DF, Powell S, Nardi A, Olney JW (1998). Clonidine potentiates the neuropathic pain-relieving action of MK-801 while preventing its neurotoxic and hyperactivity side effects. Brain Res.

[69] Afridi SK, Giffin NJ, Kaube H, Goadsby PJ (2013). A randomized controlled trial of intranasal ketamine in migraine with prolonged aura. Neurology.

[70] Alt A, Weiss B, Ogden AM, Li X, Gleason SD, Calligaro DO (2006). In vitro and in vivo studies in rats with LY293558 suggest AMPA/kainate receptor blockade as a novel potential mechanism for the therapeutic treatment of anxiety disorders. Psychopharmacology.

[71] Sang CN, Ramadan NM, Wallihan RG, Chappell AS, Freitag FG, Smith TR (2004). LY293558, a novel AMPA/GluR5 antagonist, is efficacious and well-tolerated in acute migraine. Cephalalgia.

[72] Curto M, Lionetto L, Negro A, Capi M, Fazio F, Giamberardino MA (2015). Altered kynurenine pathway metabolites in serum of chronic migraine patients. J Headache Pain.

[73] Curto M, Lionetto L, Negro A, Capi M, Perugino F, Fazio F (2015). Altered serum levels of kynurenine metabolites in patients affected by cluster headache. J Headache Pain.

[74] Schwarcz R (2004). The kynurenine pathway of tryptophan degradation as a drug target. Curr Opin Pharmacol.

[75] Zádori D, Ilisz I, Klivényi P, Szatmári I, Fülöp F, Toldi J (2011). Time-course of kynurenic acid concentration in mouse serum following the administration of a novel kynurenic acid analog. J Pharm Biomed Anal.

[76] Vécsei L, Szalárdy L, Fülöp F, Toldi J (2013). Kynurenines in the CNS: recent advances and new questions. Nat Rev Drug Discov.

[77] Szalardy L, Zadori D, Toldi J, Fulop F, Klivenyi P, Vecsei L (2012). Manipulating kynurenic acid levels in the brain - on the edge between neuroprotection and cognitive dysfunction. Curr Top Med Chem.

[78] Bohár Z, Párdutz Á, Vécsei L (2016). Tryptophan catabolites and migraine. Curr Pharm Des.

[79] Vámos E (2012). Protective compounds in animal models of trigeminal activation and neurodegeneration. Ideggyogy Sz.

[80] Messlinger K, Russo AF (2019). Current understanding of trigeminal ganglion structure and function in headache. Cephalalgia.

[81] Tuboly G, Tar L, Bohar Z, Safrany-Fark A, Petrovszki Z, Kekesi G (2015). The inimitable kynurenic acid: the roles of different ionotropic receptors in the action of kynurenic acid at a spinal level. Brain Res Bull.

[82] Knyihar-Csillik E, Mihaly A, Krisztin-Peva B, Robotka H, Szatmari I, Fulop F (2008). The kynurenate analog SZR-72 prevents the nitroglycerol-induced increase of c-fos immunoreactivity in the rat caudal trigeminal nucleus: comparative studies of the effects of SZR-72 and kynurenic acid. Neurosci Res.

[83] Park MK, Lee JH, Yang GY, Won KA, Kim MJ, Park YY (2011). Peripheral administration of NR2 antagonists attenuates orofacial formalin-induced nociceptive behavior in rats. Prog Neuro-Psychopharmacol Biol Psychiatry.

[84] Veres G, Fejes-Szabó A, Zádori D, Nagy-Grócz G, László AM, Bajtai A (2017). A comparative assessment of two kynurenic acid analogs in the formalin model of trigeminal activation: a behavioral, immunohistochemical and pharmacokinetic study. J Neural Transm (Vienna).

[85] Fejes-Szabó A, Bohár Z, Vámos E, Nagy-Grócz G, Tar L, Veres G (2014). Pre-treatment with new kynurenic acid amide dose-dependently prevents the nitroglycerine-induced neuronal activation and sensitization in cervical part of trigemino-cervical complex. J Neural Transm (Vienna).

[86] Noseda R, Borsook D, Burstein R (2017). Neuropeptides and neurotransmitters that modulate thalamo-cortical pathways relevant to migraine headache. Headache.

[87] Castle D, Robertson NP (2018). Monoclonal antibodies for migraine: an update. J Neurol.

[88] Bigal ME, Walter S, Rapoport AM (2015). Therapeutic antibodies against CGRP or its receptor. Br J Clin Pharmacol.

[89] Raffaelli B, Reuter U (2018). The biology of monoclonal antibodies: focus on calcitonin gene-related peptide for prophylactic migraine therapy. Neurotherapeutics.

[90] Vollesen AL, Benemei S, Cortese F, Labastida-Ramírez A, Marchese F, Pellesi L et al (2018) Migraine and cluster headache – the common link. J Headache Pain 19. 10.1186/s10194-018-0909-4 10.1186/s10194-018-0909-4 PMC6755613 30242519

