# Supplementary material for: Neurotransmitter and tryptophan metabolite concentration changes in the complete Freund’s adjuvant model of orofacial pain
Source: J Headache Pain. 2020 Apr 21;21(1):35. doi: 10.1186/s10194-020-01105-6 (PMC7175490; doi:10.1186/s10194-020-01105-6)
Supplement: Supplementary file 1 — Additional file 1: Table S1. Concentration levels of the measured metabolites in the cerebrospinal fluid. [file 10194_2020_1105_MOESM1_ESM.docx]

**Neurotransmitter and tryptophan metabolite concentration changes in the Complete Freund’s adjuvant model of orofacial pain**

Edina K Cseh^1,#^, Gábor Veres^1,2,#^, Tamás Körtési^1^, Helga Polyák^1^, Nikolett Nánási^1^, János Tajti^1^, Árpád Párdutz^1^, Péter Klivényi^1^, László Vécsei^1,2^, Dénes Zádori^1^*

^1^Department of Neurology, Interdisciplinary Excellence Center, Faculty of Medicine, Albert Szent-Györgyi Clinical Center, University of Szeged, Szeged, Hungary;

^2^MTA-SZTE Neuroscience Research Group, Szeged, Hungary

^#^These authors contributed equally to this work

**Table S1** Concentration levels of the measured metabolites in the cerebrospinal fluid

|  | Control group  (n = 5) | CFA 24 h  (n = 5) | CFA 48 h  (n = 4) |
| --- | --- | --- | --- |
| Cerebrospinal fluid | | | |
| Glu  (µM) | 6.08  6.04−9.60 | 9.87  4.90−16.5 | 8.61  6.13−9.96 |
| GABA  (µM) | 1.38  1.04−1.49 | 1.35  1.23−2.10 | 1.54  1.40−1.73 |
| TRP  (µM) | 1.40  0.96−1.60 | 1.32  1.24−3.77 | 1.55  1.13−1.95 |
| KYN  (µM) | < LOD | 0.21  0.13−0.22 | < LOD |
| KYNA  (nM) | 3.57  1.61−11.0 | 3.29  3.23−4.94 | 4.10  3.14−6.32 |

Results are shown as median (1^st^−3^rd^ quartile). *CFA* Complete Freund’s adjuvant*, GABA* gamma-aminobutyric acid, *Glu* glutamate, *KYN* kynurenine, *KYNA* kynurenic acid, *n* number of animals per group, *TRP* tryptophan
