# Supplementary material for: Neurotransmitter and tryptophan metabolite concentration changes in the complete Freund’s adjuvant model of orofacial pain
Source: J Headache Pain. 2020 Apr 21;21(1):35. doi: 10.1186/s10194-020-01105-6 (PMC7175490; doi:10.1186/s10194-020-01105-6)
Supplement: Supplementary file 2 — Additional file 2: Table S2. Concentration levels of the measured metabolites in the plasma samples. [file 10194_2020_1105_MOESM2_ESM.docx]

**Neurotransmitter and tryptophan metabolite concentration changes in the Complete Freund’s adjuvant model of orofacial pain**

Edina K Cseh^1,#^, Gábor Veres^1,2,#^, Tamás Körtési^1^, Helga Polyák^1^, Nikolett Nánási^1^, János Tajti^1^, Árpád Párdutz^1^, Péter Klivényi^1^, László Vécsei^1,2^, Dénes Zádori^1^*

^1^Department of Neurology, Interdisciplinary Excellence Center, Faculty of Medicine, Albert Szent-Györgyi Clinical Center, University of Szeged, Szeged, Hungary;

^2^MTA-SZTE Neuroscience Research Group, Szeged, Hungary

^#^These authors contributed equally to this work

**Table S2** Concentration levels of the measured metabolites in the plasma samples

|  | Control group  (n = 9) | CFA 24 h  (n = 9) | CFA 48 h  (n = 8) |
| --- | --- | --- | --- |
| Plasma | | | |
| TRP  (µM) | 63.9  (52.4‒78.2) | 81.4  (54.3‒88.1) | 56.4  (51.6‒76.1) |
| KYN  (µM) | 4.58  (3.29‒4.98) | 4.72  (4.45‒5.12) | 3.27  (2.83‒4.79) |
| KYNA  (nM) | 129  (120‒184) | 172  (99.9‒214) | 139  (95.0‒173) |

Results are shown as median (1^st^−3^rd^ quartile). *CFA* Complete Freund’s adjuvant*, KYN* kynurenine, *KYNA* kynurenic acid, *n* number of animals per group, *TRP* tryptophan
